# Nanotechnology-driven synergy: research effects of curcumin nanosuspension and fluconazole combination in overcoming azole resistance in *Candida albicans*

**DOI:** 10.1128/spectrum.01520-25

**Published:** 2025-10-31

**Authors:** Cheng Zhang, Jin Liu, Yunan Jiang, Shoufu Sun

**Affiliations:** 1 Shanghai Tongren Hospital, Shanghai Jiao Tong University School of Medicine, Shanghai, People’s Republic of China; University of Wisconsin-Madison, Madison, Wisconsin, USA

**Keywords:** *C. albicans*, biofilm, curcumin nanosuspension, fluconazole, synergy

## Abstract

The increasing incidence of invasive fungal infections—particularly disseminated candidiasis caused by *Candida albicans*—highlights the need for innovative therapeutic strategies. This study evaluates the antifungal activity of a curcumin nanosuspension (CNS), both alone and in combination with azole drugs, focusing on its potential to counteract drug resistance. CNS substantially improved the aqueous solubility and enabled sustained release of curcumin *in vitro*, overcoming its inherent pharmacokinetic limitations. Checkerboard assays identified synergistic effects between CNS and fluconazole against planktonic cells (fractional inhibitory concentration index [FICI]: 0.38–0.5) and early biofilms of *C. albicans* (FICI < 0.5). Morphological analyses revealed that the combination suppressed hyphal growth and disrupted biofilm integrity—critical virulence attributes of *C. albicans*. Further mechanistic observations suggested that the enhanced antifungal activity may involve facilitated drug uptake and suppression of efflux function, alongside reduced expression of adhesion-related genes (*ALS1*, *ALS3*, *HWP1*, and *EFG1*). These results underscore a promising nanotechnology-based approach to enhance the efficacy of conventional azoles through combination therapy.

**IMPORTANCE:**

This study provides a novel nanotechnology-based strategy to overcome azole resistance in *C. albicans*, a major clinical pathogen responsible for life-threatening systemic infections. By formulating curcumin into a stable nanosuspension (CNS), we significantly enhanced its solubility and bioavailability, overcoming a fundamental limitation that has hindered its clinical application. More importantly, we demonstrate that CNS acts synergistically with fluconazole, effectively restoring its efficacy against resistant planktonic cells and biofilms through dual mechanisms: increasing intracellular drug accumulation by inhibiting efflux pumps and suppressing key virulence traits including hyphal formation and adhesion. This combinatory approach not only reduces the required drug dosage and potential toxicity but also provides a promising therapeutic avenue against biofilm-associated refractory candidiasis. Our findings highlight the potential of harnessing natural product-nanocarrier systems to extend the lifespan of existing antifungals and combat drug-resistant fungal infections.

## INTRODUCTION

*Candida albicans (C. albicans*), a commensal fungus commonly found in the human microbiota, is a clinically significant opportunistic pathogen in immunocompromised individuals (1). It causes both superficial and systemic infections, particularly in organ transplant recipients, AIDS patients, and those undergoing chemotherapy, with infection-related mortality rates reaching 40% (2–4). The oral cavity is a common site of *C. albicans* colonization, with epidemiological studies reporting its presence in 45% of neonates, 45%–65% of healthy children, 30%–45% of healthy adults and up to 74% of older people (5–7). In immunocompromised hosts, colonization often progresses to oral mucositis and candidiasis. Emerging research suggests that chronic *C. albicans* infections may have oncogenic potential. Mothibe (8) reported higher fungal burdens in denture-wearing cancer patients compared to healthy controls, while Berkovits observed elevated *C. albicans* levels in patients with oral squamous cell carcinoma (9). These findings indicate a possible link between persistent fungal colonization and oral carcinogenesis that requires further investigation. Another clinical concern is the potential for localized oral infections to become systemic. Given the growing immunocompromised population worldwide and the emerging evidence of fungal oncogenicity, developing effective strategies to prevent and treat oral candidiasis has become an urgent global health priority.

The management of *C. albicans* infections depends on effective antifungal agents. Current treatments mainly include azoles (e.g., fluconazole [FLC]), polyene antifungals (e.g., amphotericin B), pyrimidine analogs (e.g., flucytosine), and echinocandins. FLC is the most frequently prescribed due to its favorable bioavailability and cost-effectiveness (10, 11). However, the rise of azole-resistant strains—often associated with prolonged prophylactic FLC use—has complicated therapeutic outcomes (12). This challenge is compounded by the limited variety of available antifungals and the increasing healthcare burden of *C. albicans* infections, highlighting an urgent need for new treatment options. In this context, natural products derived from Traditional Chinese Medicine (TCM) have gained attention as promising alternatives, offering improved bioavailability and lower toxicity. Phytochemical studies have identified several TCM-derived compounds, such as berberine from *Coptis chinensis* and baicalein from *Scutellaria baicalensis*, which enhance antifungal efficacy when combined with conventional azoles via multitarget mechanisms (13, 14). These findings underscore the potential of plant-derived medicines in overcoming antimicrobial resistance.

Curcumin (CUR), a natural compound derived from turmeric, is known for its broad-spectrum biological activities and favorable safety profile, including antibacterial, antiviral, antifungal, and anti-inflammatory properties (15, 16). Recent studies further emphasize its efficacy against multidrug-resistant fungal and bacterial pathogens (17–19), and evidence also shows that CUR inhibits the efflux pump activity of *C. albicans* (20, 21). Combining natural compounds like CUR with conventional antifungals represents a promising approach to overcoming drug resistance (22). Despite its potential, CUR’s clinical application is limited by poor aqueous solubility, low bioavailability, rapid metabolism, and inadequate blood-brain barrier penetration (23). Improving these properties—particularly solubility and dissolution kinetics—has become a crucial research focus. Particle size reduction is a key strategy for enhancing CUR’s pharmaceutical performance (24). Nanosuspensions, which are submicron colloidal dispersions stabilized with surfactants, offer a promising solution. When drug particles are reduced to submicron sizes (<1 µm), their surface area and saturation solubility increase significantly, leading to improved dissolution kinetics (25). These systems enhance drug dissolution, absorption, and bioavailability across various routes of administration (2, 26). Modern pharmaceutical techniques enable the production of uniform and stable CUR nanosuspensions (CNS), which can increase CUR’s aqueous solubility by more than 40-fold and achieve complete *in vitro* dissolution within 60 min (27). Moreover, these formulations facilitate modified release profiles, prolonging therapeutic effects (28). This advancement provides a strong foundation for overcoming the limitations of CUR in antifungal applications.

This study was aimed at combating refractory and drug-resistant *C. albicans* infections. Using a standardized *C. albicans* biofilm model, we sought to develop a CNS and evaluate its combined antifungal effects with FLC on biofilm formation. Furthermore, quantitative real-time PCR (qRT-PCR) was employed to examine the expression of key adhesion-related genes in order to elucidate the molecular mechanisms through which the combined treatment inhibits *C. albicans* biofilms. Collectively, this work offered a promising nano-based approach to overcome biofilm-associated resistance in *C. albicans*.

## RESULTS

### Characterization of a *C. albicans* biofilm model

**Fig 1:**
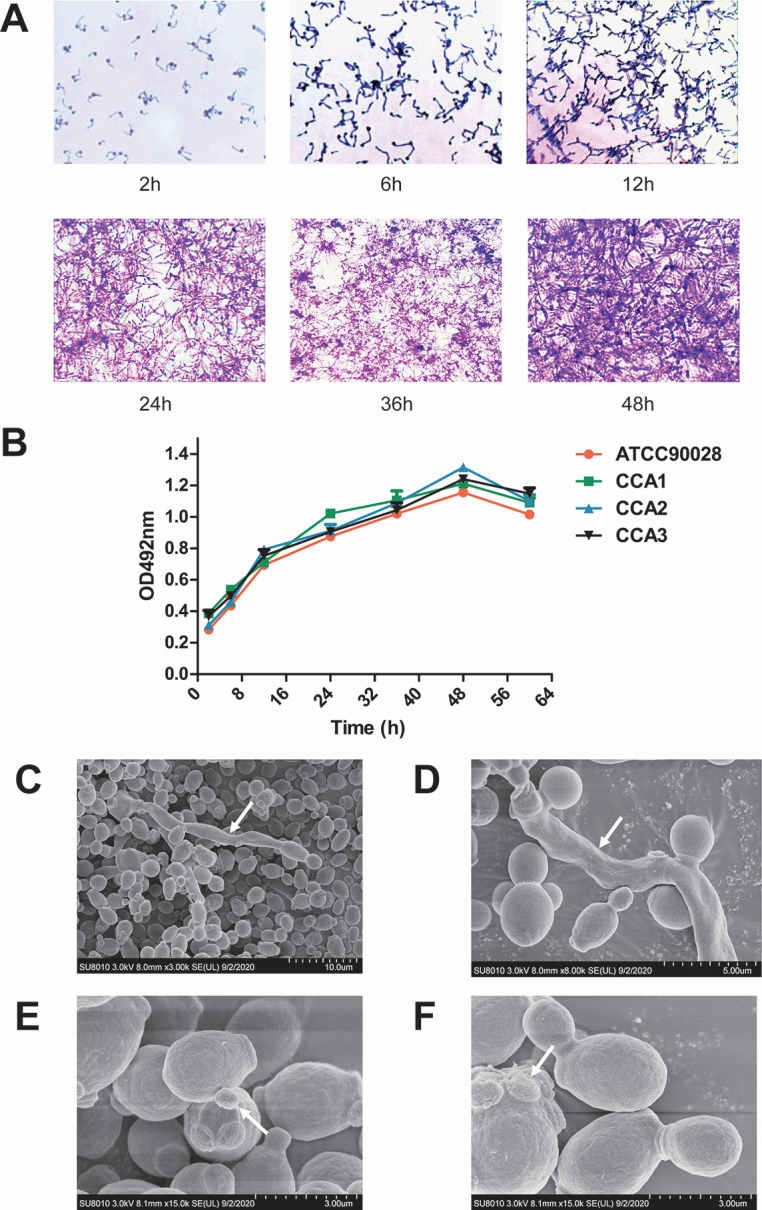
Characterization of a *C. albicans* biofilm model. (**A**) Biofilm development at different time points (2, 6, 12, 24, 36, and 48 h) was visualized by crystal violet staining in RPMI 1640 medium. (**B**) Metabolic activity of *C. albicans* biofilms was quantified using the XTT reduction assay. Data are presented as mean ± standard deviation. (C–F) SEM images illustrating typical biofilm structures: (**C**) true hyphae; (**D and E**) pseudohyphae; (**F**) blastoconidial cell showing bud scar rings and polar budding.

*C. albicans* biofilm formation was monitored over time using an inverted phase-contrast microscope. Initial yeast forms (2 h) progressively developed germ tubes and pseudohyphae by 6–12 h, followed by extensive hyphal elongation and proliferation at 24 h. Mature biofilms consisting of a dense network of yeast cells, hyphae, and extracellular matrix were observed at 48 h (Fig. 1A). The metabolic activity of the biofilms, as assessed by the XTT reduction assay, increased steadily over time and reached its peak at 48 h (Fig. 1B), confirming optimal biofilm maturation at this time point for subsequent drug testing. Scanning electron microscopy (SEM) further revealed a highly interconnected biofilm architecture with clearly distinguishable true hyphae and pseudohyphae (Fig. 1C and D). Additionally, polar budding sites of blastoconidia and variations in bud scar locations were clearly visible (Fig. 1E and F). Based on these morphological and metabolic indicators, the 48-h time point was selected as the optimal biofilm maturation stage for all subsequent antifungal drug exposure experiments.

### Properties of CNS

**Fig 2:**
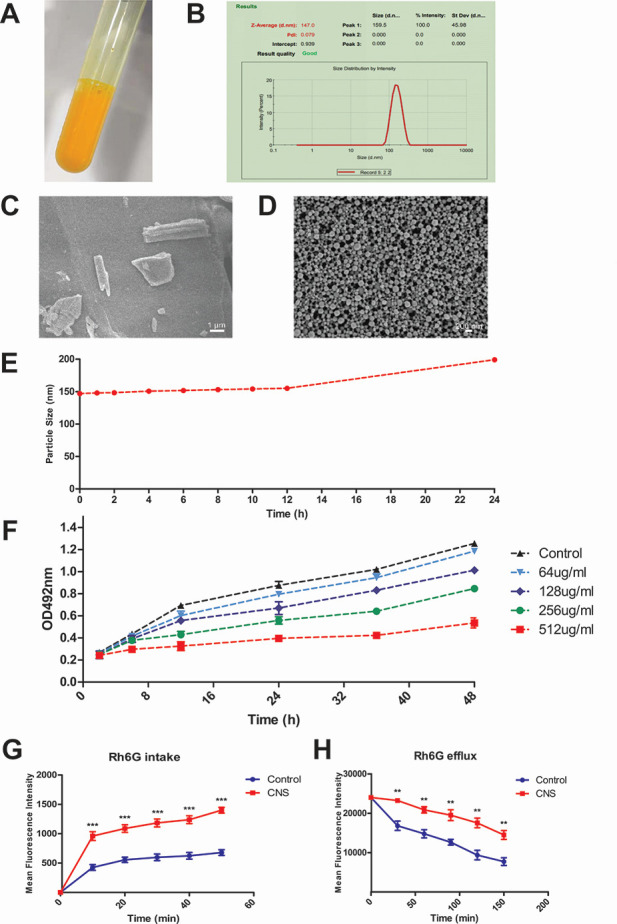
Physicochemical properties and antifungal activity of CNS. (**A**) Macroscopic appearance of the prepared CNS formulation; (**B**) particle size distribution, PDI, and zeta potential of CNS. (**C and D**) SEM images of raw CUR particles (**C**) and CNS (**D**). (**E**) Stability of CNS monitored by particle size change over 24 h. (**F**) Antifungal activity of CNS against planktonic *C. albicans* assessed by XTT assay. (**G and H**) Effect of CNS on Rh6G uptake (**G**) and efflux (**H**) in *C. albicans* measured via flow cytometry. Data are presented as mean ± SD; **P < 0.01, ***P < 0.001.

The CNS prepared as described exhibited a narrow size distribution and good dispersibility (Fig. 2A and B). The average particle size was 147.0 ± 1.304 nm, with a polydispersity index (PDI) of 0.079 ± 0.013 and a zeta potential of –28.8 ± 0.251 mV. Electron microscopy images revealed that raw CUR particles were irregular, rod-like, and microsized (Fig. 2C), whereas CNS particles were spherical, uniformly distributed, and non-aggregated, with a smooth surface morphology (Fig. 2D). The particle size observed by electron microscopy correlated well with laser particle size analysis. Stability assessment showed that the CNS remained stable within the first 12 h, after which the particle size gradually increased, reaching approximately 200 nm by 24 h (Fig. 2E). These results indicate that the CNS possessed suitable stability for subsequent pharmaceutical applications.

### MIC and SMIC₅₀ of *C. albicans*

As shown in Table 1 and Fig. 2F, CNS alone exhibited slight antifungal activity against planktonic *C. albicans* (minimum inhibitory concentration [MIC] > 256 µg/mL). However, the combination of CNS and FLC markedly reduced the MIC values against planktonic cells, with fractional inhibitory concentration index (FICI) values ranging from 0.25 to 0.5, indicating a strong synergistic effect. The combined activity of CNS and FLC was further evaluated against biofilms of four *C. albicans* strains (Table 2). CNS reduced the sessile MIC (SMIC_₅₀_) of FLC from 1,024 to 32–128 µg/mL, with FICI values between 0.25 and 0.38, demonstrating consistent synergy across all tested strains.

**TABLE 1:** MIC of CNS and FLC against planktonic cells

Strains	Planktonic cells (μg/mL)				
	MIC alone		MIC in combination		FICI (interaction)
	CNS	FLC	CNS	FLC	
ATCC90028	256	2	32	0.5	0.38
CCA1	512	4	32	1	0.31
CCA2	512	4	128	1	0.5
CCA3	512	512	128	2	0.25

**TABLE 2:** SMIC_50_ of CNS and FLC against biofilm cells

Strains	Biofilm cells (μg/mL)				
	SMIC_50_ alone		SMIC_50_ in combination		FICI (interaction)
	CNS	FLC	CNS	FLC	
ATCC90028	512	256	64	32	0.25
CCA1	1,024	512	128	64	0.25
CCA2	1,024	512	128	128	0.38
CCA3	1,024	1,024	256	128	0.38

### Uptake and efflux of rhodamine 6G

Both Rh6G and FLC are substrates of drug efflux pumps located on the cell membrane of *C. albicans*. In this study, Rh6G served as a fluorescent surrogate for FLC to monitor transport activity. Based on preliminary experiments, 128 µg/mL CNS was selected for further analysis due to its significant reduction of MIC of FLC against FLC-resistant *C. albicans* CCA3. Intracellular fluorescence intensity was significantly higher in the CNS-treated group compared to the control (Fig. 2G and H, *P* < 0.05), suggesting that CNS enhances the accumulation of Rh6G within the cells. These results imply that the synergistic antifungal effect of CNS and FLC is associated with the modulation of drug uptake and efflux mechanisms in FLC-resistant *C. albicans*.

### Inhibitory effects of CNS and FLC on *C. albicans* biofilms

**Fig 3:**
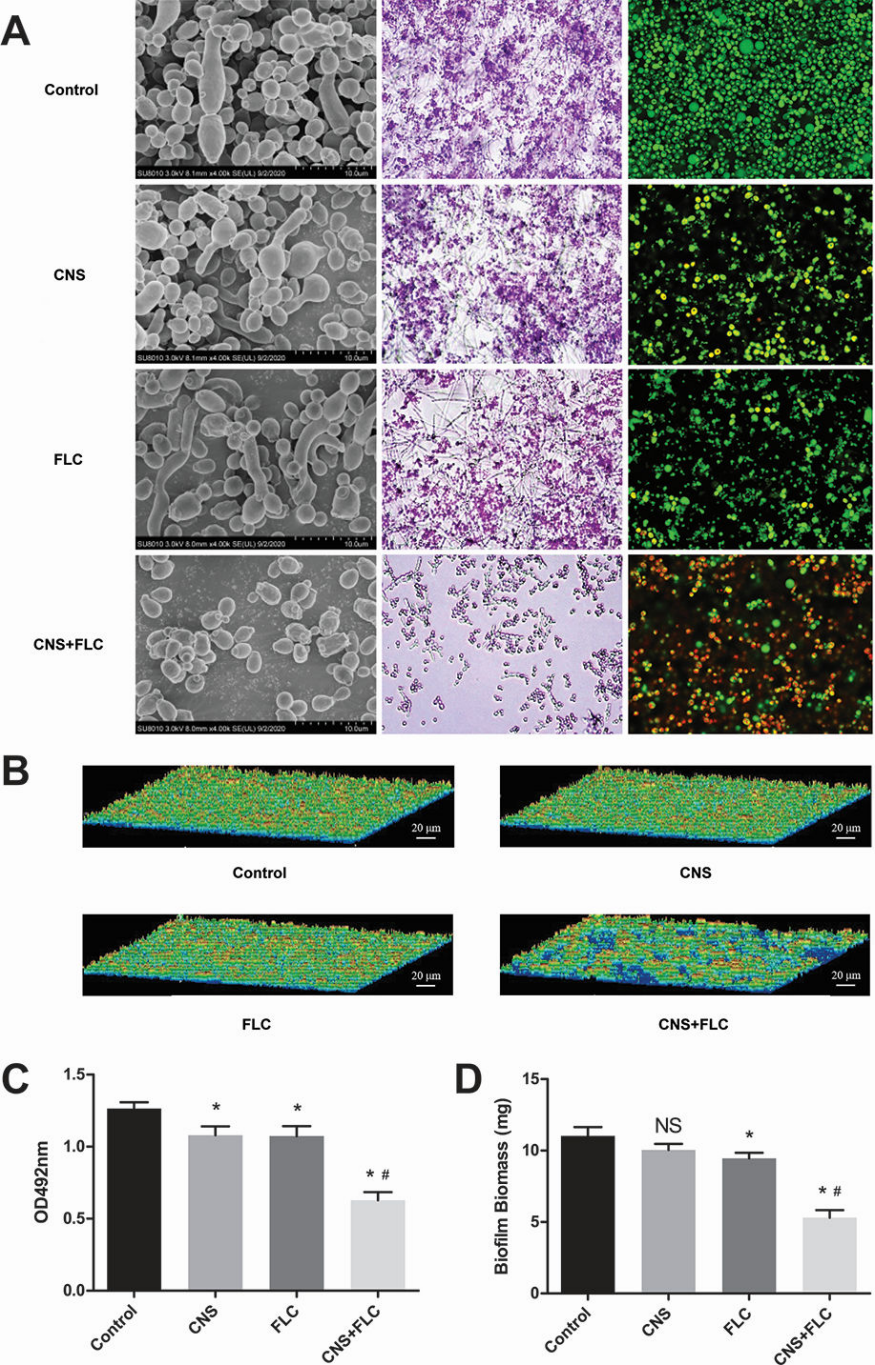
Inhibitory effects of CNS and FLC on *C. albicans* biofilms. (**A**) Representative SEM images, crystal violet staining, and CLSM micrographs of biofilms treated with control, CNS, FLC, or CNS + FLC. (**B**) Three-dimensional reconstruction of CLSM images. (**C**) Metabolic activity of biofilms measured by XTT assay. (**D**) Biofilm biomass quantified post-treatment. *P < 0.05 vs control; #*P* < 0.01 vs FLC group; NS, not significant.

SEM imaging of *C. albicans* ATCC90028 biofilms revealed distinct morphological differences among groups (Fig. 3A). The blank control group showed mature biofilms with dense hyphal networks. Treatment with CNS or FLC alone reduced hyphal density and disrupted biofilm integrity. In contrast, the combination group nearly abolished hyphal growth and biofilm formation, with only scattered cells visible. Inverted microscopy observations correlated well with SEM results. While control biofilms exhibited dense, structured hyphal mats, single-agent treatments resulted in thinner, monolayer architectures. The combination group showed only isolated cells with no organized biofilm. Confocal laser scanning microscopy (CLSM) imaging using SYTO9 and propidium iodide staining further confirmed the enhanced efficacy of the combination treatment. Control biofilms showed vigorous green fluorescence (viable cells), with minimal red signal. Single treatments moderately reduced viability, whereas the combination group presented markedly reduced green fluorescence and increased red staining, indicating extensive cell death. 3D reconstruction of biofilms corroborated these findings (Fig. 3B). The combination group displayed severely disrupted biofilm topography compared to the structured architectures seen in the control and single-treatment groups.

The XTT assay revealed that the combined treatment significantly reduced metabolic activity compared to both the blank control and single-agent groups (Fig. 3C, *P* < 0.05). Additionally, biomass quantification by dry weight measurement showed that the combination group exhibited a pronounced reduction in biofilm mass relative to all other groups (Fig. 3D, *P* < 0.05), demonstrating effective inhibition of biofilm formation by CNS and FLC.

### Quantification of adhesion and biofilm-related gene expression by qRT-PCR

**Fig 4:**
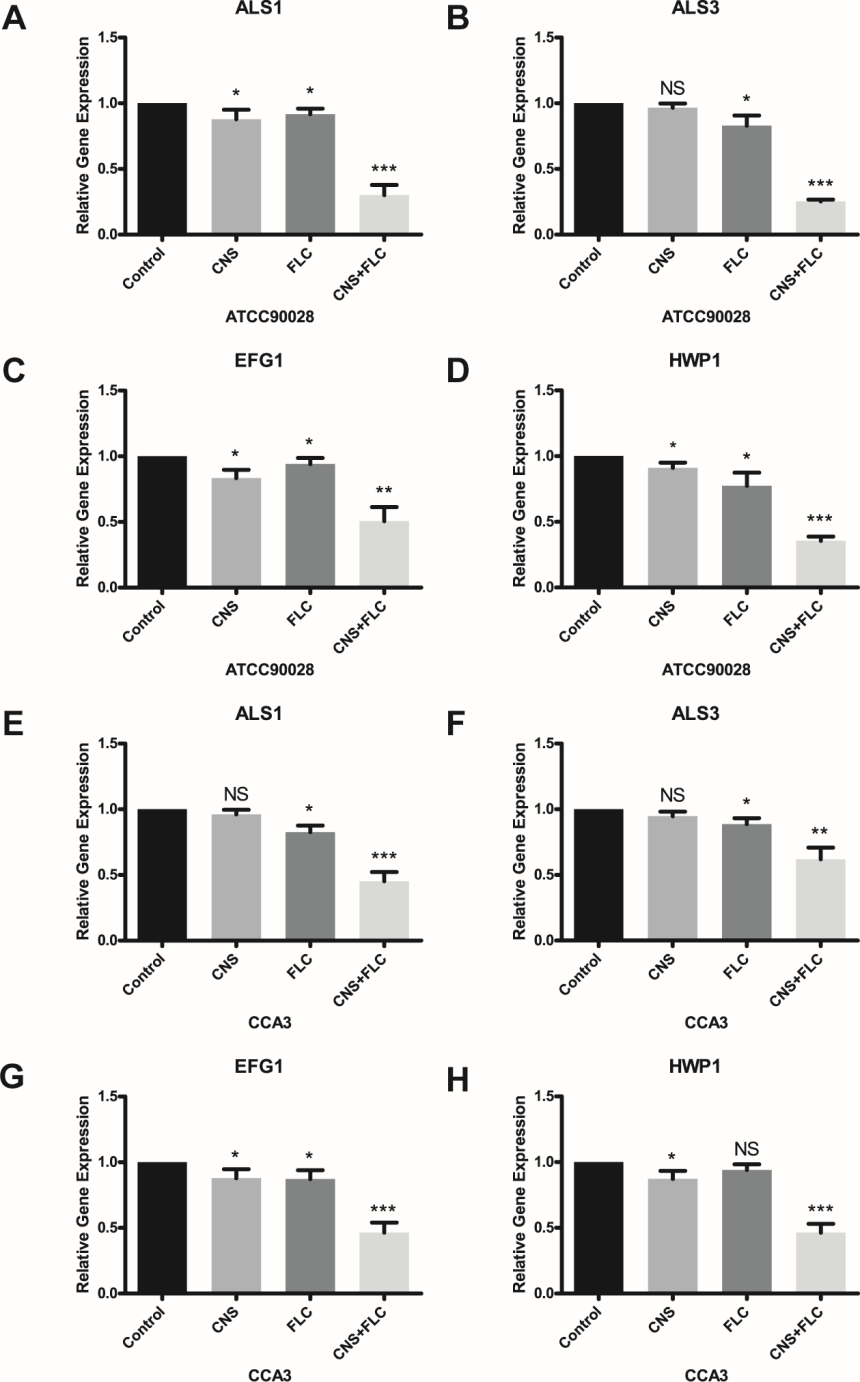
Effects of CNS and FLC on the expression of adhesion and biofilm-related genes in *C. albicans*. (**A–D**) Expression of ALS1 (**A**), ALS3 (**B**), EFG1 (**C**), and HWP1 (**D**) in ATCC90028 under different treatments. (**E–H**) Expression of ALS1 (**E**), ALS3 (**F**), EFG1 (**G**), and HWP1 (**H**) in CCA3 under different treatments, as determined by qPCR. *P < 0.05, **P < 0.01, ***P < 0.001; NS, not significant.

The expression of genes related to adhesion and biofilm formation (*ALS1*, *ALS3*, *HWP1*, and *EFG1*) was quantified using qRT-PCR. All target genes exhibited downregulated expression after drug treatment. While CNS or FLC alone caused slight reductions in expression compared to the control, the combination of CNS and FLC resulted in significant downregulation (Fig. 4). These results suggest that the combined treatment strongly suppresses genes associated with adhesion and hyphal development in *C. albicans*.

## DISCUSSION

Over recent decades, the incidence of fungal infections has risen significantly, primarily attributable to the widespread use of broad-spectrum antibiotics and a growing population of immunocompromised individuals—including cancer patients, organ transplant recipients, those living with HIV, and the elderly (29). Among these pathogens, *C. albicans* is the most prevalent, capable of causing infections ranging from superficial skin disorders to life-threatening systemic conditions, posing a particular threat to immunocompromised hosts (3, 29). FLC, a triazole antifungal agent, remains a first-line treatment for *C. albicans* infections owing to its efficacy and favorable safety profile. However, prolonged and extensive use of FLC has led to a marked increase in antifungal resistance (30). Moreover, *C. albicans* biofilms, capable of forming on both biological and inert surfaces, function as physical barriers that confer inherent resistance to most antifungal drugs (31). These challenges underscore the urgent need to develop novel therapeutic strategies to combat drug-resistant *C. albicans*.

Natural products derived from TCM represent a valuable source for developing novel antifungal agents. Numerous plant-derived compounds have shown potential as either standalone antifungals or synergists to enhance conventional antifungal drugs (32). Among them, CUR has been extensively studied for its broad pharmacological properties, including anticancer, antibacterial, anti-inflammatory, and anti-aging effects, as demonstrated in various *in vitro*, *in vivo*, and clinical settings (33). For instance, Lee and Lee (34) reported that CUR exerts antifungal activity by disrupting the fungal plasma membrane, while Garcia-Goes et al. (35) showed that it can sensitize clinically resistant strains of *C. albicans* to azole drugs *in vitro*. Additionally, CUR-based photodynamic therapy has been shown to effectively inactivate *C. albicans* biofilms (36). Despite these promising properties, the clinical translation of CUR is hindered by its poor aqueous solubility, low bioavailability, and unfavorable pharmacokinetic profile (33).

To overcome these limitations, various strategies have been developed to enhance the bioavailability, bioactivity, and physicochemical properties of CUR (37). In particular, nanotechnology offers promising approaches for designing novel antibacterial, antifungal, and entomotoxic agents (38). Through nanoparticle formulation, nanosystems achieve enhanced stability, biocompatibility, and improved bioactivity (39–41). In this study, the CNS exhibited spherical, well-dispersed, and non-aggregated morphology, accompanied by favorable dispersibility, solubility, and stability. Previous studies reported MIC values of CUR against various microorganisms ranging from 250 to 2,000 µg/mL (42). Consistent with these findings, CNS alone showed modest antifungal activity, with MICs between 256 and 512 µg/mL. Sharma et al. (43) demonstrated that combining CUR with azoles or polyenes reduced the MIC values by 10- to 35-fold against clinical isolates of *C. albicans*. Similarly, Dong et al. (44) developed several CUR derivatives that restored FLC efficacy against FLC-resistant *C. albicans*. In line with these reports, our results revealed that while CNS or FLC alone only partially reduced biofilm thickness and hyphal formation, the combination of CNS and FLC significantly suppressed *C. albicans* biofilm development, disrupted architectural integrity, and largely restricted cells to the yeast form.

The resistance of *C. albicans* to azole antifungals, such as FLC, is frequently associated with enhanced efflux pump activity (45). CUR inhibits efflux pumps in *C. albicans* primarily by downregulating the expression of ABC transporters such as *CDR1* through post-transcriptional disruption of Hsp90 and also directly competes with substrates like Rh6G to block efflux activity (20, 21). Our results demonstrated that CNS significantly enhanced Rh6G accumulation and suppressed its efflux in the resistant strain CCA3, indicating potent inhibition of drug transport pump activity. The agglutinin-like sequence (*ALS*) genes play essential roles in the pathogenicity of *C. albicans. ALS1* encodes adhesins that mediate binding to endothelial and epithelial cells during biofilm formation (46), while *ALS3* promotes hyphal development and cellular adhesion (47). *HWP1* facilitates fungal attachment to host cells and strengthens biofilm integrity (48), and *EFG1* regulates morphogenesis, hyphal formation, and virulence (49). In our study, we observed that treatment with CNS or FLC alone could slightly downregulate the expression of four key biofilm-related genes (*ALS1*, *ALS3*, *HWP1*, and *EFG1*). In this study, treatment with CNS or FLC alone only slightly downregulated the expression of these key biofilm-related genes (*ALS1*, *ALS3*, *HWP1*, and *EFG1*). In contrast, the combination of CNS and FLC led to significant downregulation of all four genes. This synergistic transcriptional inhibition likely contributed to the profound suppression of *C. albicans* biofilm formation observed herein.

Although our results indicate that CUR modulates efflux pump activity and enhances FLC efficacy, the precise molecular mechanisms—particularly the regulation of fungal transporters and related signaling pathways—require further investigation. Additionally, although initial experiments demonstrated good stability of CNS, its physical and biological properties must be rigorously evaluated over extended periods and under various storage conditions to ensure pharmaceutical suitability. Furthermore, comprehensive biocompatibility assessments using *in vitro* cell assays and *in vivo* animal models are essential to verify the safety and therapeutic potential of CNS before clinical translation. Besides the aforementioned considerations, it should be noted that the fungal resuscitation and pre-culture protocol employed in this study, while optimized for biofilm formation based on established methods, involved specific temperature shifts that may influence cellular physiology. This technical aspect warrants further validation in future research to determine its potential effects on the observed phenotypes.

In summary, this study demonstrates that CNS significantly inhibits the growth of *C. albicans* and enhances the antifungal efficacy of FLC. Synergistic effects between CNS and FLC were visually confirmed through SEM and laser confocal microscopy, showing substantial disruption of biofilm integrity and cellular viability. Mechanistic insights suggest that the synergy likely stems from the suppression of hyphal development, impairment of cell adhesion, and inhibition of efflux pump activity. Further investigation is necessary to fully clarify the molecular mechanisms underlying this synergistic antifungal action.

## MATERIALS AND METHODS 

### Strains, reagents, and growth conditions

Four specimens of *C. albicans* strains, including one laboratory strain (ATCC 90028, American Type Culture Collection, USA), and three clinical strains (CCA1, CCA2, and CCA3), were obtained from the Fungal Laboratory of Huashan Hospital, Fudan University (Shanghai, China). Approval from the Institutional Review Board of Huashan Hospital and written informed consents were obtained from these patients. Among these, CCA3 was confirmed as an FLC-resistant clinical strain by the Fungal Laboratory of Fudan University. Fungal cells were stored in Sabouraud dextrose agar (SDA) medium at −4°C and reactivated by subculturing in the fresh SDA medium at 28°C for 48 h prior to use. Overnight cultures were prepared in Yeast Extract-Peptone-Dextrose (YPD) broth containing 1% (wt/vol) yeast extract, 2% (wt/vol) peptone, and 2% (wt/vol) glucose. Single colonies were inoculated into 30 mL fresh YPD medium and incubated at 32°C with orbital shaking (160 rpm) for 15 h following previously described protocols (36). Cell pellets were obtained by centrifugation at 3,000 rpm for 10 min at 4°C, followed by two washes with sterile phosphate-buffered saline (PBS, pH 7.4). The washed cells were resuspended in RPMI-1640 medium (supplemented with L-glutamine, buffered with sodium bicarbonate) (Gibco, Grand Island, NY, USA) and adjusted to a final density of 2.0 × 10⁶ cells/mL using an improved Neubauer hemocytometer. The CUR was generously provided by the School of Pharmacy, Fudan University (Shanghai). FLC was purchased from Sigma Co., Ltd (Sigma-Aldrich, MO, USA).

*C. albicans* cell suspensions (2 × 10⁶ CFU/mL) were inoculated into sterile 96-well plates at 100 µL per well and cultured for 2–48 h (2, 6, 12, 24, 36, and 48 h) at 37°C. After each time point, the cells were fixed with 100 µL of formaldehyde for 20 min, followed by staining with 0.001% crystal violet for 10 min. The plates were then gently rinsed with PBS until the wash solution became clear. Biofilm morphology was observed using an inverted phase-contrast microscope. Additionally, cell viability was assessed by laser confocal microscopy, structural features were examined via SEM, and metabolic activity was evaluated using the XTT reduction assay.

### XTT reduction assays

In the XTT reduction assays, menaphone solution (0.4 mM) was prepared by dissolving menaphone powder in acetone and stored at 4℃. XTT powder was mixed with PBS solution (pH 7.4) to obtain a 1 mg/mL test solution, which was stored at −70℃. Then, 2 µL of 0.4 mM menaphone solution, 40 µL of 1 mg/mL XTT solution, and 158 µL of PBS solution containing 200 mM glucose were thoroughly mixed and filtered through a 0.22 µm sterile filter. Biofilms of *C. albicans* strains, including the standard strain ATCC90028 and clinical strains CCA1, CCA2, and CCA3, were cultured for varying durations (2–60 h). After removing the supernatant and rinsing twice with PBS, 200 µL of the XTT mixed solution was added to each well. The biofilms were then incubated in the dark at 37℃ for 3 h. A microplate analyzer was used to detect the absorbance at 492 nm, which indicated the metabolic activity of the biofilms. All experiments were performed in triplicate and repeated three times.

### SEM observation

*C. albicans* cell suspensions (2 × 10⁶ CFU/mL) were inoculated into 24-well plates containing RPMI-1640 medium and a cover glass at the bottom, followed by incubation at 37°C for 48 h. The cover glass was gently washed three times with sterile PBS and fixed with 2.5% glutaraldehyde at 4°C for 72 h. The fixed biofilm was then rinsed three times with 0.1 mol/L phosphate buffer (15 min per wash). Subsequently, the samples were post-fixed with 1% osmium tetroxide for 3 h and rinsed again three times with phosphate buffer. Dehydration was performed using a graded ethanol series (50%, 70%, 80%, 90%, and 100%; 20 min per step). The biofilm was then treated with a 1:1 mixture of absolute ethanol and ethyl acetate for 30 min, followed by pure ethyl acetate for another 30 min. Finally, the dried samples were sputter-coated with gold and observed under a scanning electron microscope (S-2460N SEM, Hitachi, JP).

### Preparation and physical characterization of CNS

A CNS with a target particle size of 200 nm was prepared as follows: 0.012 g of CUR was weighed and dissolved in 0.4 mL of acetone under sonication to form the organic phase. This solution was then transferred into 4.6 mL of 0.1% HPMC E5 solution under continuous magnetic stirring. The organic solvent was removed using a rotary evaporator. The final concentration of CUR was adjusted to 2.6 mg/mL with 0.1% HPMC E5 solution.

For particle size and PDI analysis, the CNS was diluted 40-fold with ultrapure water. A 500 µL aliquot of the diluted suspension was loaded into the sample chamber of a laser particle size analyzer (Zetasizer, Malvern Panalytical Ltd., UK). Measurements were performed at 25°C after a 60 s equilibration time. Three replicates were conducted per sample, and data were processed using Zetasizer Software Version 7.11. Zeta potential was measured using a dedicated dip cell, ensuring the liquid level covered the electrodes. For SEM imaging, 50 µL of the 40-fold diluted CNS was deposited on a 50 nm filter membrane, air-dried, washed three times, and allowed to dry naturally. The membrane was then trimmed, mounted on a stub using conductive tape, and sputter-coated with gold for 60 s before observation at 30,000× magnification under an emission voltage of 5.0 kV. To evaluate stability, 3 mL of CNS was stored at room temperature. Samples (200 µL) were collected at 1, 2, 4, 6, 8, 10, 12, and 24 h, diluted 40-fold, and analyzed for particle size and PDI using the same laser particle size analyzer.

### Determination of MIC in planktonic cells

The MIC of CNS and FLC, both alone and in combination, against planktonic *C. albicans* cells were determined using the broth microdilution method according to CLSI document M27-A3 (50). Checkerboard assays were conducted with CNS concentrations ranging from 1 to 512 µg/mL and FLC from 1 to 32 µg/mL. *C. albicans* suspensions (2 × 10³ CFU/mL) in RPMI-1640 medium were inoculated into 96-well plates, with various drug concentrations applied per well, including a blank control. After 24 h of incubation at 37°C in the dark, the MIC₈₀ was defined as the lowest drug concentration that resulted in 80% growth inhibition compared to the blank control. Absorbance at 492 nm (OD₄₉₂) was measured using a BioTek Synergy two spectrophotometer (USA). All tests were performed in triplicate and independently repeated three times.

The interaction between FLC and CNS was assessed using the FICI, calculated as follows: FICI = (MIC of drug A in combination / MIC of drug A alone) + (MIC of drug B in combination / MIC of drug B alone). An FICI of ≤0.5 was interpreted as synergy, >0.5 to ≤4.0 as no interaction, and >4.0 as antagonism.

### Determination of SMIC₅₀ against *C. albicans* biofilms

The SMIC₅₀ of CNS and FLC against *C. albicans* biofilms was evaluated with concentrations ranging from 1 to 1024 µg/mL for CNS and 1 to 512 µg/mL for FLC. *C. albicans* cell suspensions (2 × 10⁶ CFU/mL) were inoculated into 96-well plates containing RPMI-1640 medium and incubated for 48 h at 37°C to allow biofilm formation. Various concentrations of the drugs were introduced upon inoculation, with a blank control included. After incubation, biofilm viability was assessed using the XTT reduction assay, and OD₄₉₂ was measured with the BioTek Synergy two spectrophotometer. The SMIC₅₀ was defined as the lowest drug concentration that inhibited 50% of biofilm metabolic activity relative to the blank control. All experiments were conducted in triplicate and repeated three times. The combined effect of CNS and FLC on biofilms was also evaluated using the checkerboard method and interpreted via the FICI model as described above.

### Rhodamine 6G uptake and efflux assays

The FLC-resistant *C. albicans* strain CCA3 (1 × 10⁵ CFU/mL) was cultured in YPD liquid medium at 35°C with shaking (180 rpm) for 18–19 h. Cells were collected by centrifugation (3,000 × *g*, 5 min) and washed three times with glucose-free PBS. The cell density was adjusted to 1 × 10⁷ CFU/mL using a hemocytometer, and cells were de-energized in glucose-free PBS for 2 h at 35°C with gentle shaking (50 rpm).

For the uptake assay: Rh6G (10 µM final concentration) and CNS (128 µg/mL final concentration) were added to the cell suspensions. Controls received Rh6G alone. Intracellular fluorescence intensity was measured at 10 min intervals using flow cytometry (BD FACSVerse, USA; ex/em: 488/530 nm) until the signal stabilized.

For the efflux assay: De-energized cells were pre-loaded with 10 µM Rh6G at 35°C for 50 min and immediately chilled in an ice-water bath for 10 min to inhibit transport. After three washes with cold PBS to remove extracellular dye, cells were resuspended in PBS containing either CNS (128 µg/mL) or Rh6G alone (control). Fluorescence was measured at 30-min intervals via flow cytometry until a plateau was reached.

### Morphological analysis of biofilms by CLSM and SEM

The inhibitory effects of CNS and FLC, both alone and in combination, on *C. albicans* biofilms were assessed using CLSM and SEM. *C. albicans* strain ATCC90028 cell suspensions (2 × 10⁶ CFU/mL) were inoculated into laser confocal culture dishes or 24-well plates containing cover slips and cultured in RPMI-1640 medium. The following treatment groups were evaluated: (i) blank control, (ii) FLC 32 µg/mL, (iii) CNS 64 µg/mL, and (iv) FLC +CNS (32 µg/mL + 64 µg/mL). After 48 h of incubation at 37°C, biofilms were processed for observation.

For CLSM, a LIVE/DEAD Fungal Light Yeast Viability Kit (Molecular Probes, Invitrogen, USA) was used to distinguish viable and non-viable cells. The staining solution, containing PI and SYTO-9 at a ratio of 1.5 µL:1.5 µL in 1 mL saline, was applied to each dish (400 µL/dish) and incubated for 20 min at room temperature in the dark. Images were acquired at excitation/emission wavelengths of 490/635 nm (PI) and 480/500 nm (SYTO-9).

For SEM, biofilms grown on cover slips were fixed, dehydrated, and critical-point dried as previously described. Samples were then sputter-coated with gold and imaged under a scanning electron microscope.

### Quantitative assessment of biofilm viability and biomass

The same treatment groups and incubation conditions as above were used for quantitative assays. *C. albicans* ATCC90028 suspensions (1 × 10⁶ cells/mL) were incubated with CNS and/or FLC for 48 h at 37°C.

For metabolic activity assessment, the XTT assay was performed. After discarding the supernatant and washing twice with PBS, 200 µL of XTT mixture was added to each well, followed by incubation for 3 h. Absorbance at 492 nm was measured using a microplate reader.

For biomass quantification, the dry weight method was employed. Biofilms were digested with 0.25% trypsin for 10 min, collected by centrifugation (2,000 rpm, 15 min, 4°C), and washed three times with PBS. The pellet was vacuum-dried and weighed. The biofilm weight was calculated by subtracting the weight of the empty EP tube.

### qRT-PCR

The expression levels of biofilm-related genes (*ALS1*, *ALS3*, *HWP1*, and *EFG1*) were evaluated in *C. albicans* strains ATCC90028 and CCA3 after 48 h of drug treatment under the following conditions: ATCC90028: (i) control, (ii) FLC 32 µg/mL, (iii) CNS 64 µg/mL, and (iv) FLC + CNS; CCA3: (1) control, (ii) FLC 128 µg/mL, (iii) CNS 256 µg/mL, and (4) FLC + CNS. Total RNA was extracted using 1 mL of TRIzol Reagent. cDNA was synthesized from 1 µg of RNA. qPCR was performed using a SYBR Green master mix (Takara, Japan) with gene-specific primers (see Table 3). Relative gene expression was calculated using the 2^–ΔΔCt method (51).

**TABLE 3:** Primers used in this study

Gene	Forward primer (5′→3′)	Reverse primer (5′→3′)
16sRNA	AGCCAGCGAGTATAAGCCTT	AAGGGCAGGGACGTAATCAA
ALS1	CATGTACGTTGCTATCCAGGC	CAAATCGGAGGTTGTGCTGT
ALS3	TTCTCGTCCTCATTACACCAACC	ATGAAGTTGCAGATGGGGCT
EFG1	ACAAGTGCTCCTAGTGGTGC	GGACTAGTGGTGGAACCTGC
HWP1	ACAAGGAATTCGGAAATTCTGACG	CGGTTGTGAGCCATTAGGGT

### Statistical analysis

All statistical analyses were performed using SPSS 19.0 (IBM, USA). Continuous data with normal distribution are presented as mean ± standard deviation. Differences between two groups were compared using independent samples *t*-test. A *P*-value of less than 0.05 was considered statistically significant.

## References

[1] Boral H, Metin B, Döğen A, Seyedmousavi S, Ilkit M. 2018. Overview of selected virulence attributes in Aspergillus fumigatus, Candida albicans, Cryptococcus neoformans, Trichophyton rubrum, and Exophiala dermatitidis. Fungal Genet Biol 111:92–107. doi:10.1016/j.fgb.2017.10.008 29102684

[2] Suleyman G, Alangaden GJ. 2021. Nosocomial fungal infections: epidemiology, infection control, and prevention. Infect Dis Clin North Am 35:1027–1053. doi:10.1016/j.idc.2021.08.002 34752219

[3] Dadar M, Tiwari R, Karthik K, Chakraborty S, Shahali Y, Dhama K. 2018. Candida albicans - Biology, molecular characterization, pathogenicity, and advances in diagnosis and control - An update. Microb Pathog 117:128–138. doi:10.1016/j.micpath.2018.02.028 29454824

[4] Thambugala KM, Daranagama DA, Tennakoon DS, Jayatunga DPW, Hongsanan S, Xie N. 2024. Humans vs. Fungi: an overview of fungal pathogens against humans. Pathogens 13:426. doi:10.3390/pathogens13050426 38787278 PMC11124197

[5] Patel M. 2022. Oral cavity and Candida albicans: colonisation to the development of infection. Pathogens 11:335. doi:10.3390/pathogens11030335 35335659 PMC8953496

[6] Zaremba ML, Daniluk T, Rozkiewicz D, Cylwik-Rokicka D, Kierklo A, Tokajuk G, Dabrowska E, Pawińska M, Klimiuk A, Stokowska W, Abdelrazek S. 2006. Incidence rate of Candida species in the oral cavity of middle-aged and elderly subjects. Adv Med Sci 51 Suppl 1:233–236. 17458099

[7] Kullberg BJ, Arendrup MC. 2015. Invasive candidiasis. N Engl J Med 373:1445–1456. doi:10.1056/NEJMra1315399 26444731

[8] Mothibe JV, Patel M. 2017. Pathogenic characteristics of Candida albicans isolated from oral cavities of denture wearers and cancer patients wearing oral prostheses. Microb Pathog 110:128–134. doi:10.1016/j.micpath.2017.06.036 28655563

[9] Berkovits C, Tóth A, Szenzenstein J, Deák T, Urbán E, Gácser A, Nagy K. 2016. Analysis of oral yeast microflora in patients with oral squamous cell carcinoma. Springerplus 5:1257. doi:10.1186/s40064-016-2926-6 27536540 PMC4974209

[10] Nett JE, Andes DR. 2016. Antifungal agents: spectrum of activity, pharmacology, and clinical indications. Infect Dis Clin North Am 30:51–83. doi:10.1016/j.idc.2015.10.012 26739608

[11] Silva LJ, Silva CR, Sá LG, Barroso FD, Cândido TM, Queiroz HA, Almeida Moreira LE, Baccallini OV, Cavalcanti BC, Silva J, Marinho ES, Moraes MO, Neto JB, Júnior HV. 2022. Antifungal activity of dexamethasone against fluconazole-resistant Candida albicans and its activity against biofilms. Future Microbiol 17:607–620. doi:10.2217/fmb-2021-0146 35411812

[12] Sanguinetti M, Posteraro B, Lass-Flörl C. 2015. Antifungal drug resistance among Candida species: mechanisms and clinical impact. Mycoses 58 Suppl 2:2–13. doi:10.1111/myc.12330 26033251

[13] Liu S, Hou Y, Chen X, Gao Y, Li H, Sun S. 2014. Combination of fluconazole with non-antifungal agents: a promising approach to cope with resistant Candida albicans infections and insight into new antifungal agent discovery. Int J Antimicrob Agents 43:395–402. doi:10.1016/j.ijantimicag.2013.12.009 24503221

[14] Huang S, Cao YY, Dai BD, Sun XR, Zhu ZY, Cao YB, Wang Y, Gao PH, Jiang YY. 2008. In vitro synergism of fluconazole and baicalein against clinical isolates of Candida albicans resistant to fluconazole. Biol Pharm Bull 31:2234–2236. doi:10.1248/bpb.31.2234 19043205

[15] Tsuda T. 2018. Curcumin as a functional food-derived factor: degradation products, metabolites, bioactivity, and future perspectives. Food Funct 9:705–714. doi:10.1039/C7FO01242J 29206254

[16] Peng S, Li Z, Zou L, Liu W, Liu C, McClements DJ. 2018. Improving curcumin solubility and bioavailability by encapsulation in saponin-coated curcumin nanoparticles prepared using a simple pH-driven loading method. Food Funct 9:1829–1839. doi:10.1039/c7fo01814b 29517797

[17] Kumar A, Dhamgaye S, Maurya IK, Singh A, Sharma M, Prasad R. 2014. Curcumin targets cell wall integrity via calcineurin-mediated signaling in Candida albicans. Antimicrob Agents Chemother 58:167–175. doi:10.1128/AAC.01385-13 24145527 PMC3910804

[18] Packiavathy I, Priya S, Pandian SK, Ravi AV. 2014. Inhibition of biofilm development of uropathogens by curcumin - an anti-quorum sensing agent from Curcuma longa. Food Chem 148:453–460. doi:10.1016/j.foodchem.2012.08.002 24262582

[19] Tyagi P, Singh M, Kumari H, Kumari A, Mukhopadhyay K. 2015. Bactericidal activity of curcumin I is associated with damaging of bacterial membrane. PLoS One 10:e0121313. doi:10.1371/journal.pone.0121313 25811596 PMC4374920

[20] Lee YS, Chen X, Widiyanto TW, Orihara K, Shibata H, Kajiwara S. 2022. Curcumin affects function of Hsp90 and drug efflux pump of Candida albicans. Front Cell Infect Microbiol 12:944611. doi:10.3389/fcimb.2022.944611 36237434 PMC9551236

[21] Sharma M, Manoharlal R, Shukla S, Puri N, Prasad T, Ambudkar SV, Prasad R. 2009. Curcumin modulates efflux mediated by yeast ABC multidrug transporters and is synergistic with antifungals. Antimicrob Agents Chemother 53:3256–3265. doi:10.1128/AAC.01497-08 19470507 PMC2715616

[22] Puumala E, Fallah S, Robbins N, Cowen LE. 2024. Advancements and challenges in antifungal therapeutic development. Clin Microbiol Rev 37:e0014223. doi:10.1128/cmr.00142-23 38294218 PMC10938895

[23] Anand P, Kunnumakkara AB, Newman RA, Aggarwal BB. 2007. Bioavailability of curcumin: problems and promises. Mol Pharm 4:807–818. doi:10.1021/mp700113r 17999464

[24] Wang R, Zou L, Yi Z, Zhang Z, Zhao M, Shi S. 2023. PLGA nanoparticles loaded with curcumin produced luminescence for cell bioimaging. Int J Pharm 639:122944. doi:10.1016/j.ijpharm.2023.122944 37044226

[25] Kocbek P, Baumgartner S, Kristl J. 2006. Preparation and evaluation of nanosuspensions for enhancing the dissolution of poorly soluble drugs. Int J Pharm 312:179–186. doi:10.1016/j.ijpharm.2006.01.008 16469459

[26] Göke K, Lorenz T, Repanas A, Schneider F, Steiner D, Baumann K, Bunjes H, Dietzel A, Finke JH, Glasmacher B, Kwade A. 2018. Novel strategies for the formulation and processing of poorly water-soluble drugs. Eur J Pharm Biopharm 126:40–56. doi:10.1016/j.ejpb.2017.05.008 28532676

[27] Li X, Yuan H, Zhang C, Chen W, Cheng W, Chen X, Ye X. 2016. Preparation and in-vitro/in-vivo evaluation of curcumin nanosuspension with solubility enhancement. J Pharm Pharmacol 68:980–988. doi:10.1111/jphp.12575 27283220

[28] Wang Y, Wang C, Zhao J, Ding Y, Li L. 2017. A cost-effective method to prepare curcumin nanosuspensions with enhanced oral bioavailability. J Colloid Interface Sci 485:91–98. doi:10.1016/j.jcis.2016.09.003 27657837

[29] Lee Y, Puumala E, Robbins N, Cowen LE. 2021. Antifungal drug resistance: molecular mechanisms in Candida albicans and beyond. Chem Rev 121:3390–3411. doi:10.1021/acs.chemrev.0c00199 32441527 PMC8519031

[30] Whaley SG, Berkow EL, Rybak JM, Nishimoto AT, Barker KS, Rogers PD. 2016. Azole antifungal resistance in Candida albicans and emerging non-albicans Candida species. Front Microbiol 7:2173. doi:10.3389/fmicb.2016.02173 28127295 PMC5226953

[31] Bonhomme J, d’Enfert C. 2013. Candida albicans biofilms: building a heterogeneous, drug-tolerant environment. Curr Opin Microbiol 16:398–403. doi:10.1016/j.mib.2013.03.007 23566895

[32] Zhong H, Han L, Lu RY, Wang Y. 2022. Antifungal and immunomodulatory ingredients from traditional Chinese medicine. Antibiotics (Basel) 12:48. doi:10.3390/antibiotics12010048 36671249 PMC9855100

[33] Kotha RR, Luthria DL. 2019. Curcumin: biological, pharmaceutical, nutraceutical, and analytical aspects. Molecules 24:2930. doi:10.3390/molecules24162930 31412624 PMC6720683

[34] Lee W, Lee DG. 2014. An antifungal mechanism of curcumin lies in membrane-targeted action within Candida albicans. IUBMB Life 66:780–785. doi:10.1002/iub.1326 25380239

[35] Garcia-Gomes AS, Curvelo JAR, Soares RMA, Ferreira-Pereira A. 2012. Curcumin acts synergistically with fluconazole to sensitize a clinical isolate of Candida albicans showing a MDR phenotype. Med Mycol 50:26–32. doi:10.3109/13693786.2011.578156 21539505

[36] Ma J, Shi H, Sun H, Li J, Bai Y. 2019. Antifungal effect of photodynamic therapy mediated by curcumin on Candida albicans biofilms in vitro. Photodiagnosis Photodyn Ther 27:280–287. doi:10.1016/j.pdpdt.2019.06.015 31233886

[37] Racz LZ, Racz CP, Pop L-C, Tomoaia G, Mocanu A, Barbu I, Sárközi M, Roman I, Avram A, Tomoaia-Cotisel M, Toma V-A. 2022. Strategies for improving bioavailability, bioactivity, and physical-chemical behavior of curcumin. Molecules 27:6854. doi:10.3390/molecules27206854 36296447 PMC9608994

[38] Varshan GSA, Namasivayam SKR. 2025. A critical review on sustainable formulation of anti-quorum sensing compounds using nanotechnology principles against Candida albicans. BioNanoSci 15. doi:10.1007/s12668-024-01685-6

[39] Thabet AF, Galal OA, Gao S, Tuda M, Fujita R, Hino M, Miksanek JR, K.c. B, Kishimura A, El–Samahy MF, Mousa KM. 2025. Oxidative effects of foliar-applied silica, titania, and silver nanoparticles on the leafminer, with additional studies of silica nanoparticle impacts on survival and development time. Plant Nano Biology 13:100164. doi:10.1016/j.plana.2025.100164

[40] Raj LFAA, Annushrie A, Namasivayam SKR. 2025. Anti bacterial efficacy of photo catalytic active titanium di oxide (TiO2) nanoparticles synthesized via green science principles against food spoilage pathogenic bacteria. Microbe 7:100331. doi:10.1016/j.microb.2025.100331

[41] Sivasuriyan KS, Namasivayam SKR, Rajendran S, Ganesan Subbulakshmi AV. 2025. Biocompatible chitosan-starch bio-composite fabricated with Mukia maderaspatana metabolites: preparation and evaluation for enhanced potential pharmacological activities. Next Materials 8:100911. doi:10.1016/j.nxmate.2025.100911

[42] Rai M, Ingle AP, Pandit R, Paralikar P, Anasane N, Santos CAD. 2020. Curcumin and curcumin-loaded nanoparticles: antipathogenic and antiparasitic activities. Expert Rev Anti Infect Ther 18:367–379. doi:10.1080/14787210.2020.1730815 32067524

[43] Sharma M, Manoharlal R, Negi AS, Prasad R. 2010. Synergistic anticandidal activity of pure polyphenol curcumin I in combination with azoles and polyenes generates reactive oxygen species leading to apoptosis. FEMS Yeast Res 10:570–578. doi:10.1111/j.1567-1364.2010.00637.x 20528949

[44] Dong HH, Wang YH, Peng XM, Zhou HY, Zhao F, Jiang YY, Zhang DZ, Jin YS. 2021. Synergistic antifungal effects of curcumin derivatives as fungal biofilm inhibitors with fluconazole. Chem Biol Drug Des 97:1079–1088. doi:10.1111/cbdd.13827 33506609

[45] Holmes AR, Cardno TS, Strouse JJ, Ivnitski-Steele I, Keniya MV, Lackovic K, Monk BC, Sklar LA, Cannon RD. 2016. Targeting efflux pumps to overcome antifungal drug resistance. Future Med Chem 8:1485–1501. doi:10.4155/fmc-2016-0050 27463566 PMC5827819

[46] Hoyer LL, Green CB, Oh SH, Zhao X. 2008. Discovering the secrets of the Candida albicans agglutinin-like sequence (ALS) gene family--a sticky pursuit. Med Mycol 46:1–15. doi:10.1080/13693780701435317 17852717 PMC2742883

[47] von Ranke NL, Bello ML, Cabral LM, Castro HC, Rodrigues CR. 2018. Molecular modeling and dynamic simulations of agglutinin-like family members from Candida albicans: new insights into potential targets for the treatment of candidiasis. J Biomol Struct Dyn 36:4352–4365. doi:10.1080/07391102.2017.1417159 29241420

[48] Ene IV, Bennett RJ. 2009. Hwp1 and related adhesins contribute to both mating and biofilm formation in Candida albicans. Eukaryot Cell 8:1909–1913. doi:10.1128/EC.00245-09 19837954 PMC2794213

[49] Connolly LA, Riccombeni A, Grózer Z, Holland LM, Lynch DB, Andes DR, Gácser A, Butler G. 2013. The APSES transcription factor Efg1 is a global regulator that controls morphogenesis and biofilm formation in Candida parapsilosis. Mol Microbiol 90:36–53. doi:10.1111/mmi.12345 23895281 PMC3912905

[50] CLSI. 2017. Reference method for broth dilution antifungal susceptibility testing of yeasts. 4th ed. Clinical and Laboratory Standards Institute.

[51] Livak KJ, Schmittgen TD. 2001. Analysis of relative gene expression data using real-time quantitative PCR and the 2(-Delta Delta C(T)) Method. Methods 25:402–408. doi:10.1006/meth.2001.1262 11846609

